# A Patient With Single Coronary Artery and Pectus Excavatum Presenting With Non-ST Elevation Myocardial Infarction

**DOI:** 10.7759/cureus.42163

**Published:** 2023-07-19

**Authors:** Vsevolod Tabachnikov, Amir Aker, Ibrahim Naoum, Salim Bashir, Yuval Avidan

**Affiliations:** 1 Cardiology, Carmel Medical Center, Haifa, ISR

**Keywords:** percutaneous coronary intervention, non-st elevation myocardial infarction, pectus excavatum, lipton anomaly, single coronary artery

## Abstract

We report the case of a 51-year-old male with pectus excavatum (PEX) who presented with stress-related chest pain as a symptom of acute non-ST elevation myocardial infarction. Coronary angiography (CAG) revealed a suspected single coronary artery (SCA) anatomy with diffuse atherosclerotic narrowing, without evidence of other coronary ostia in the aortic root. The diagnosis was confirmed on cardiac computed tomography (CCTA) as the SCA of the R-I type by Lipton classification. The percutaneous coronary intervention was performed with good angiographic results and resolution of symptoms.

## Introduction

Single coronary artery (SCA) is rarely seen in the population and usually presents with other congenital abnormalities (tetralogy of Fallot, truncus arteriosus, pulmonary atresia, etc.). Nevertheless, in rare cases, it may present as an acute coronary syndrome in adults without other diagnosed congenital abnormalities. Up until the last decades, it was mostly revealed during an autopsy, but with the wide spreading of coronary angiography, the anomaly detection rate has grown significantly. However, according to existing literature, their occurrence still remains very low (0.02-0.04%) [1]. In 1979, Lipton proposed the classification of SCA anomalies according to the origination of the artery from the left or right coronary sinus and its further course [2].

## Case presentation

**Figure 1:**
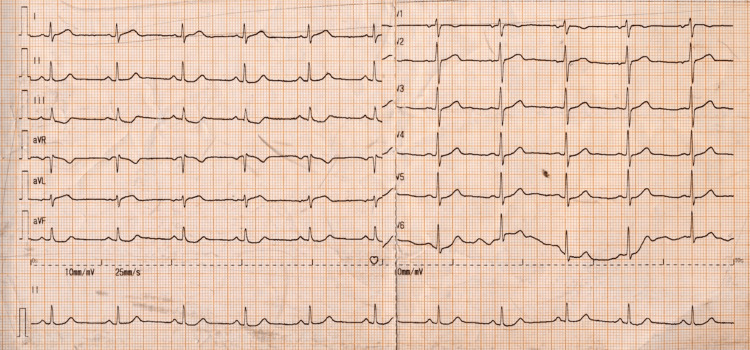
12-lead electrocardiogram at presentation showing sinus rhythm with T-wave inversion in the inferior leads (II, III, aVF).

A 51-year-old male with multiple cardiovascular risk factors (hypertension, dyslipidemia, obesity) presented with two hours of chest pain, nausea, and sweating, triggered by emotional stress. His vital signs were unremarkable. Physical examination was notable for pectus excavatum (PEX) and chest wall tenderness. The 12-lead electrocardiogram (ECG) revealed 1.5 mm ST segment depression in inferior leads (Figure 1).

**Figure 2:**
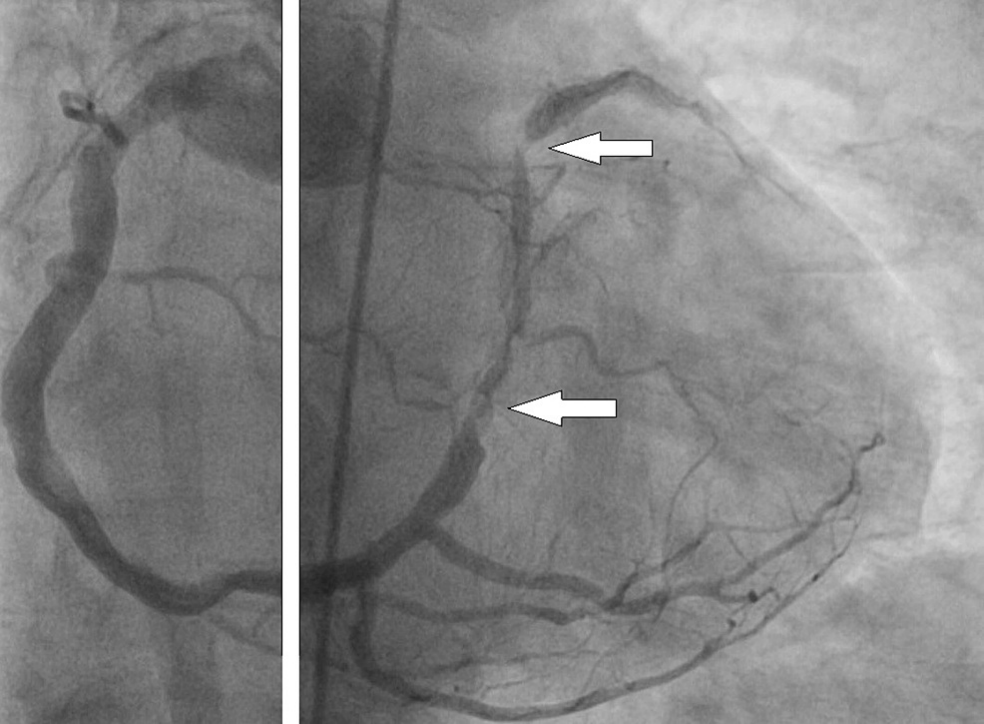
Coronary angiographic images prior to percutaneous coronary intervention showing right anterior oblique (RAO) cranial view of a single right coronary artery (RCA) which gives off the left anterior descending and circumflex analogs. There are flow-limiting lesions with severe luminal narrowing in the distal segments. Due to the relatively slow filling of the artery by contrast media, the image is split in two.

Laboratory workup showed significant elevation in Troponin T - 596.5 ng/L (reference range <13). Echocardiographic examination showed no signs of reduced contractility or evidence of structural heart disease. Coronary angiography (CAG) demonstrated that the right coronary artery is following its normal anatomical course, which provides the posterolateral branches (circumflex artery analog) and then continues to the anterior basal surface of the heart and makes terminal anterior descending artery (Figure 2).

**Figure 3:**
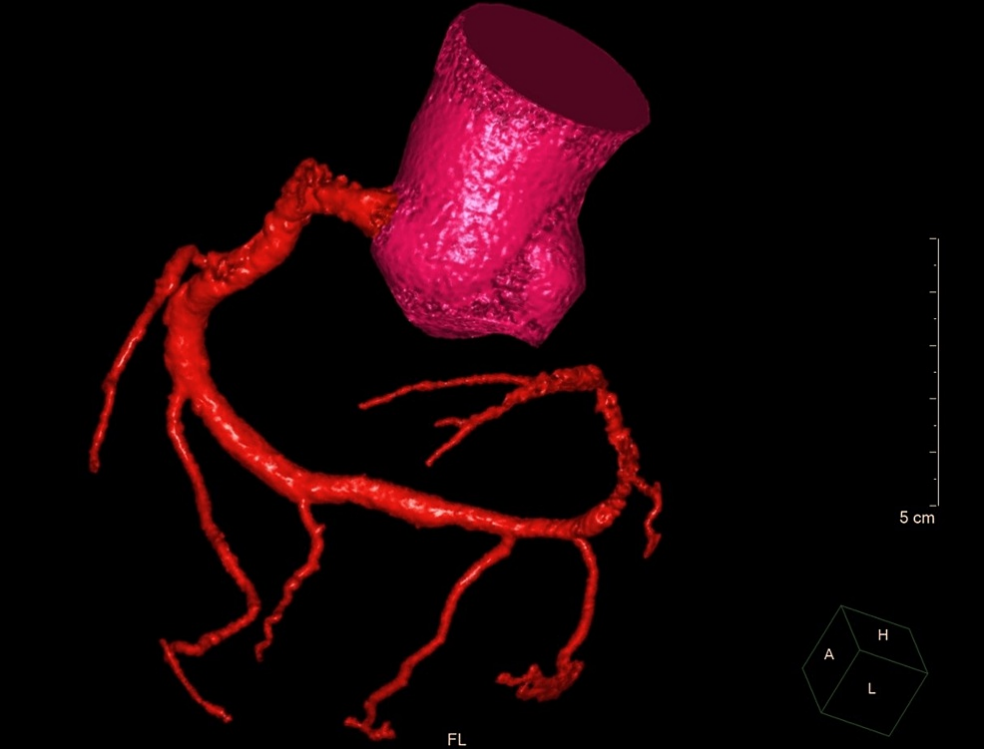
3D CT reconstruction of ascending aorta and single coronary artery CT: computed tomography

Besides anatomical aspects, 80% narrowing was diagnosed in the circumflex branch analog and 90% narrowing in the proximal and middle left anterior descending branch analog. The patient was referred to coronary computed tomography angiography (CCTA) which confirmed the angiographic diagnosis of a single right coronary artery, type R-I, according to Lipton classification (Figure 3).

**Figure 4:**
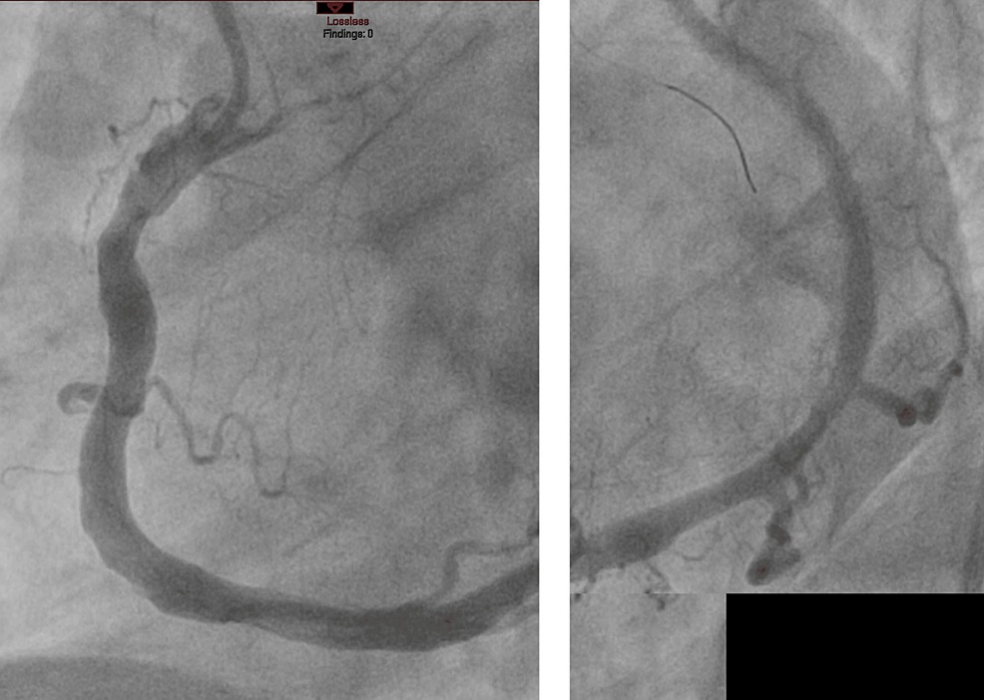
Caudal angiographic images demonstrating a single coronary artery after angioplasty with good angiographic result. Due to the relatively slow filling of the artery by contrast media, the image is split in two.

The patient finally underwent percutaneous coronary intervention with the implantation of three drug-eluting stents with acceptable angiographic results and subsequent resolution of symptoms (Figure 4).

He was discharged in good condition on optimal medical therapy. Follow-up at six and 12 months was uneventful.

## Discussion

The prognosis of patients with SCA remains uncertain and varies from benign (with no decreased life expectancy) to sudden cardiac death. Ischemia may be also present as a result of anatomical anomalies, such as acute take-off angle, slit-like orifice, etc., that makes CCTA exceptionally useful not just for confirming the absence of a second coronary artery (which may not be seen in CAG due to ostial occlusion), but also for risk assessment [3]. Additionally, displacement of the coronary ostium and increased blood flow in SCA may lead to endothelial damage and further acceleration of the atherosclerotic processes. It is also reported, that signs of CAD can be seen in about 15% of patients with SCA in the absence of significant coronary disease due to abnormal coronary anatomy [1].

R-I type of Lipton anomaly usually takes a benign course because the SCA originates from the right coronary sinus and is not susceptible to lateral compression. Therefore, there is no need for surgical correction of such pathology [1]. However, it may result in difficulties in approaching the lesion during the coronary intervention and increase the risk of massive infarction in the case of proximal or middle occlusion due to the absence of collaterals.

Another intriguing aspect is the ECG findings. Despite the fact that CAG in our case identified a substantial coronary disease in both the circumflex artery and terminal anterior descending artery analogs, in a patient with acute presentation and substantial cardiac biomarker elevation, the ECG depicted only a minimal ST segment depression in the inferior leads. Ischemic ECG changes in patients with Lipton anomaly are poorly studied and require further evaluation. Since no previous ECG has been documented, we cannot exclude the presence of these abnormalities at baseline.

Another important aspect of this report relates to PEX, a fairly prevalent chest wall defect in which the sternum and costal cartilages are posteriorly displaced. PEX can be part of genetic syndromes such as Marfan syndrome, Noonan syndrome, or other coexisting cardiac malformations. Scoliosis is common [4]. The assessment of chest pain in a patient with PEX might be challenging. First, in older individuals, various symptoms have been attributed to PEX including exercise intolerance, palpitations, and anterior chest wall discomfort. Second, echocardiographic assessment can be difficult. The right side of the heart may be impacted by the anatomical variations of the sternum and the vertebral column, substantially complicating echocardiographic assessment [4]. Finally, an association between PEX and coronary anomalies is unknown. To our knowledge, this is the first reported case of these two congenital abnormalities coexisting in a patient.

## Conclusions

The evaluation of chest pain in patients with PEX may be clinically and echocardiographically challenging. The diagnosis of myocardial infarction in patients with SCA may also be difficult, as ECG may underestimate the degree of ischemia. Such patients should be treated with extreme caution and a high index of suspicion.
